# Low-Cost Optical Mapping Systems for Panoramic Imaging of Complex Arrhythmias and Drug-Action in Translational Heart Models

**DOI:** 10.1038/srep43217

**Published:** 2017-02-27

**Authors:** Peter Lee, Conrado J. Calvo, José M. Alfonso-Almazán, Jorge G. Quintanilla, Francisco J. Chorro, Ping Yan, Leslie M. Loew, David Filgueiras-Rama, José Millet

**Affiliations:** 1 Essel Research and Development Inc., Toronto, Canada; 2 ITACA, Universitat Politècnica de València, Valencia, Spain; 3 Spanish National Cardiovascular Research Center, Carlos III (CNIC), Madrid, Spain; 4 Departments of Physiology and Cardiology, Universitat de València, Clinic Hospital - INCLIVA, Valencia, Spain; 5 Centro de Investigación Biomédica en Red de Enfermedades Cardiovasculares (CIBERCV), Spain; 6 Instituto de Investigación Sanitaria del Hospital Clínico San Carlos (IdISSC), Madrid, Spain; 7 Richard D. Berlin Center for Cell Analysis and Modeling, University of Connecticut School of Medicine, Farmington, CT, USA

## Abstract

Panoramic optical mapping is the primary method for imaging electrophysiological activity from the entire outer surface of Langendorff-perfused hearts. To date, it is the only method of simultaneously measuring multiple key electrophysiological parameters, such as transmembrane voltage and intracellular free calcium, at high spatial and temporal resolution. Despite the impact it has already had on the fields of cardiac arrhythmias and whole-heart computational modeling, present-day system designs precludes its adoption by the broader cardiovascular research community because of their high costs. Taking advantage of recent technological advances, we developed and validated low-cost optical mapping systems for panoramic imaging using Langendorff-perfused pig hearts, a clinically-relevant model in basic research and bioengineering. By significantly lowering financial thresholds, this powerful cardiac electrophysiology imaging modality may gain wider use in research and, even, teaching laboratories, which we substantiated using the lower-cost Langendorff-perfused rabbit heart model.

Optical mapping is a high-speed fluorescence imaging method that uses fluorescent dyes to measure cardiac and neuronal electrophysiological parameters such as transmembrane voltage, intracellular free calcium and cell-to-cell communication. In the cardiac electrophysiology field, this method has enabled researchers to study properties of both healthy and diseased heart tissue at high spatial and temporal resolution^1^,^2^. Coupled with the use of drugs to perturb component cell and tissue activity, this method has helped provide mechanistic insights into how life-threatening arrhythmias arise and how they are sustained. Furthermore, whole-heart computer modeling and simulation studies may also be validated with optical mapping data, leading to the design and development of rational antiarrhythmic therapies *in silico*^3^.

A critical limitation of using optical mapping to study fibrillation on the epicardial surface of a Langendorff-perfused heart is that a camera can only take 2D images from one point-of-view. If one wants to detect ventricular ectopic beats or track spiral waves out of the one field-of-view afforded by a single camera, a panoramic optical mapping system is needed^1^,^2^. Panoramic imaging systems either use multiple cameras, mirrors, or both to simultaneously image the globe-shaped heart from multiple points-of-view. System configurations providing from two to four points-of-view have been developed by several groups^4^,^5^,^6^,^7^. Two points-of-view allows one to image the anterior (front) and posterior (back) of the heart while four points-of-view yields overlapping fields-of-view and more complete spatial information. All such systems have enabled the visualization and quantitative analysis of complex arrhythmias.

Despite its impact on the field of cardiac arrhythmias, panoramic optical mapping continues to be a prohibitively expensive procedure, limiting its widespread use. Currently, the highest cost components are the high-speed fluorescence imaging cameras. Considering that the cost of the cameras most commonly used in the field is ~$40,000 USD each, constructing a panoramic imaging system utilizing four cameras may put the use of this powerful method out of reach to many research programs standing to gain from it. Add to this the cost of the optics, light sources, computer, electronics, mechanical and optomechanical components, it is easy to conceive of the total system cost rising above ~$180,000 USD.

The cameras used for optical mapping must meet certain minimum requirements to generate data of sufficient quality. Using standard fluorescent dyes, the photodetectors of the camera should be capable of recording action potentials and calcium transients with adequately high signal-to-noise ratios (SNRs) under nonfibrillatory and fibrillatory rhythms. Furthermore, these recordings must be achieved at minimal spatial and temporal resolution to capture propagation of the activation front and to describe wave patterns. For pig hearts, studies have shown negligible signal content above 100 Hz in the temporal domain and below 3 × 3 mm in the spatial domain^8^. Hence, a camera with a frame-rate/sampling-frequency of at least 200 Hz and resolution of at least 50 × 50 pixels will surpass these minimal resolution limits (conservatively assuming heart dimensions of 15 × 15 cm). If the goal of the imaging system is to capture all spatiotemporal phenomena on the epicardial surface of the heart, then the camera must be able to detect the highest frequency events at the requisite spatial resolution. Among the common animal models used for obtaining translational cardiac electrophysiology data (ex. rabbits and pigs), ventricular fibrillation in the rabbit heart represents the highest frequency events, reaching as high as ~30 Hz^9^,^10^. Although a minimum sampling frequency of ~60 Hz is needed, a sampling frequency of at least 300 Hz will preserve the general morphology of action potentials and calcium transients.

In this study, we describe three optical mapping systems built around a collection of four Wide-VGA USB3.0 CMOS cameras (~$600 USD each at the time of writing) and one 2.2 Megapixel USB3.0 CMOS camera (~$1200 USD at the time of writing). Although the imaging systems described here were mainly validated with pig hearts, the systems were designed to be used with animal models ranging in size from the adult rabbit to the adult pig (or human). The most costly system described here is a four-camera optical mapping system providing four overlapping fields-of-view^11^. The total cost of this system, including all the optics, light sources, custom-built computer, electronics, mechanical and optomechanical components, was less than the ~$40,000 USD typically spent for one standard optical mapping camera system. Although some applications will certainly require such high-performance cameras, the systems described here represent inexpensive alternatives capable of yielding data of sufficient quality for arrhythmia research.

We now present the design and validation of three optical mapping systems. The first system uses four cameras to image the transmembrane voltage from the entire epicardial surface of the heart from four equally-spaced points-of-view. The second system uses four cameras to simultaneously image transmembrane voltage and intracellular free calcium from the anterior (two cameras) and posterior (two cameras) surface of the heart. Lastly, the third system uses just one high spatial resolution camera and multiple mirrors to image transmembrane voltage from the anterior and posterior surface of the heart. Besides meeting the minimal signal quality and spatiotemporal resolution requirements, we designed the imaging systems to be capable of making continuous long-duration recordings, which is not a common feature of panoramic optical mapping systems but has been proven to be important in the study of ventricular fibrillation^12^,^13^. In terms of illumination, we built all light-sources around modern high-power light-emitting-diodes (LEDs), driving costs down further^1^,^2^. Besides the custom-machined mechanical components, all other components used are readily obtainable off-the-shelf (see **Materials and Methods**).

## Results

### Optical Mapping System 1

**Figure 1:**
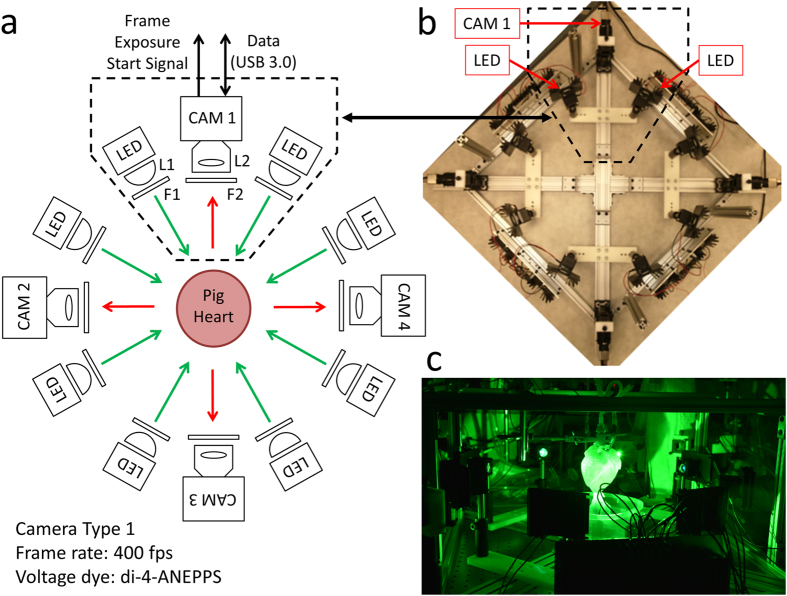
Optical Mapping System 1: Layout (Langendorff-Perfused Pig Heart). (**a**) System schematic showing key components (see text for details). One camera and two LED light sources are used to image the heart from each point-of-view (green arrows: excitation light, red arrows: fluorescence emission light). (**b**) Bird’s-eye view of the imaging and illumination subsystems outlined in (**a**). (**c**) A picture of part of the system during an experimental run. The illuminated pig heart is visible at the center of the picture.

The system shown in Fig. 1 utilizes four high-speed Wide-VGA USB3.0 CMOS cameras spaced 90° around the isolated heart on the horizontal plane, providing a 360° view of the heart ventricles^11^. Although not implemented here, a surface scanner can be used to generate a 3D surface reconstruction of the epicardial surface, enabling the fluorescence signals collected from the four cameras to be mapped onto a geometric model of the heart surface. Each camera (CAM 1 – CAM 4 in Fig. 1a) is configured to record 120 × 160 superpixels (4 × 4 binning mode; 4 × 4 pixels per superpixel) at 400 frames-per-second (fps). All cameras are controlled by a single custom-built computer via USB3.0 interfaces. The frame exposure start signal generated by each camera allows one to record when each camera captures a frame. Although each camera is triggered to start at the same time, the inherent variation between camera hardware clocks can lead to slight timing differences between cameras over long duration recordings. The recorded sequence of frame exposure start signals from each camera allows one to deal with any such drifts during post-processing of the image sequences. We found the drift of a few milliseconds over tens of seconds of recording to be negligible relative to the electrophysiological phenomena observed in this study.

As shown in Fig. 1, two LED light sources are mounted on the left and right side of each camera. The LEDs are mounted to heat sinks and the emitted light lensed and filtered to provide illumination on all imaged surfaces of the dye-loaded heart. The off-the-shelf potentiometric dye di-4-ANEPPS was used because of its popularity, ease of loading, brightness and large fractional fluorescence change during cardiac action potentials^1^,^2^,^14^,^15^.

**Figure 2:**
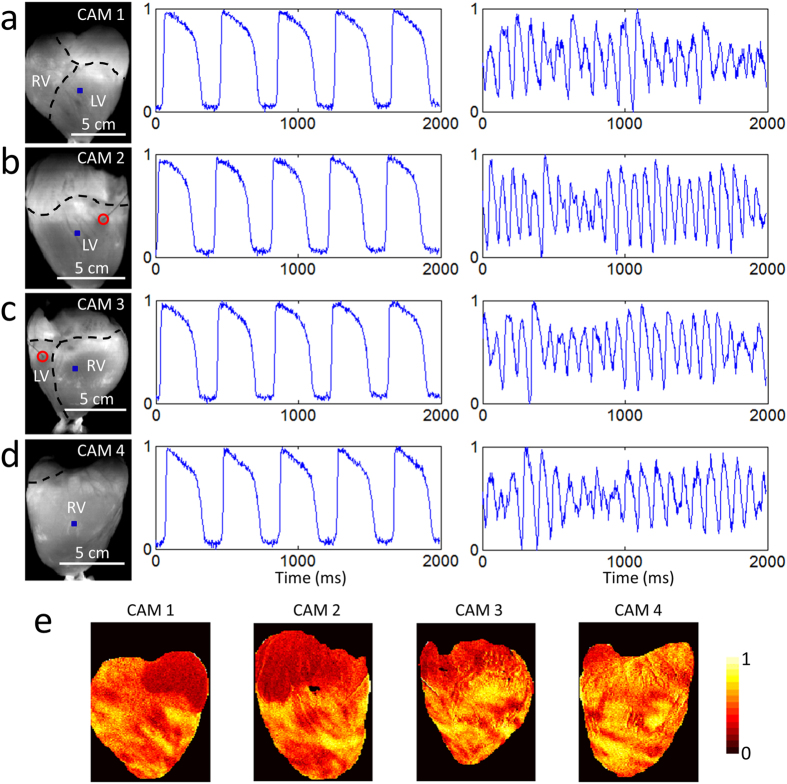
Optical Mapping System 1: Sample Data (Langendorff-Perfused Pig Heart). Normalized fluorescence signals from the (**a**) anterior, (**b**) left lateral, (**c**) posterior and (**d**) right lateral points-of-view of the heart during electrical pacing (middle panel) and ventricular fibrillation (right panel), taken from the blue-square regions shown (left panel). In the left panels, the right ventricle, left ventricle and atria are demarcated by black dashed lines and the red circle indicates the location of electrical point stimulation. Each of the four cameras can be independently positioned and rotated to adjust and optimize the position of the heart in its field-of-view. The varying scale bars in the left panels reflects these adjustments. Signals are in arbitrary fluorescence units. (**e**) Normalized fluorescence intensity map (colorbar shown) at one time point during ventricular fibrillation.

Figure 2a–d shows sample raw data, unfiltered in time. The heart was imaged while being electrically paced (Supplementary Video 1) and during ventricular fibrillation (Supplementary Video 2). The SNR was sufficient for quantitative analysis for at least one hour of experimentation. For example, during pacing, the SNRs for CAM 1, CAM 2, CAM 3 and CAM 4 were ~49, ~52, ~50, and ~46, respectively. These sample values were calculated using the signals shown in Fig. 2a–d and the formula SNR = (AP Amplitude)/(SD during diastolic intervals), where AP: action potential and SD: standard deviation^16^. Figure 2e shows a normalized transmembrane voltage fluorescence intensity maps at one time point during ventricular fibrillation, illustrating irregular electrical activity.

**Figure 3:**
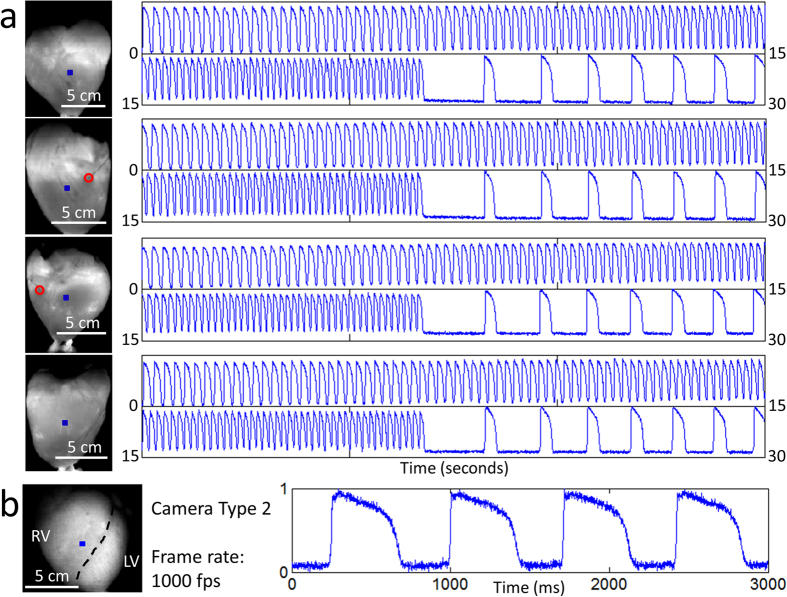
Optical Mapping System 1: Long-Duration and 1 kHz Recordings (Langendorff-Perfused Pig Heart). (**a**) Samples of long-duration recordings from the same heart shown in Fig. 2. The 30 second segments show normalized fluorescence signals from the blue-square regions (left panel) at increasing pacing frequency. At ~22 seconds, the pacing was stopped and the heart returned to sinus rhythm. In the left panels, the red circle indicates the location of electrical point stimulation. (**b**) Normalized fluorescence signals from the blue-square region (left panel) on the surface of another heart taken at higher spatial and temporal resolution. In the left panel, the black dashed line demarcates the right ventricle and left ventricle. Signals are in arbitrary fluorescence units.

Figure 3a presents samples of long-duration recordings that were taken simultaneously from all four cameras. In the 30 second segments shown, the heart was paced at increasing frequency in an attempt to induce ventricular fibrillation (at ~22 seconds, we stopped pacing). In this particular experiment, we failed to induce fibrillation and the heart returned to sinus rhythm. Although most of the proof-of-principle recordings in this study were 20–30 seconds in duration, we have tested the system’s recording capability out to a minimum of 60 seconds.

Although a frame rate of 400 fps is more than adequate to capture the highest frequency components of the action potential signal in the pig epicardium, some applications may require more temporal resolution. As a demonstration, we replaced one of the Wide-VGA USB3.0 CMOS cameras with the 2.2 Megapixel USB3.0 CMOS camera used in **Optical Mapping System 3** (described later). This more expensive camera provides higher spatial and temporal resolution while maintaining similarly sized pixels. Configuring the camera to record 160 × 220 superpixels (4 × 4 binning mode; 4 × 4 pixels per superpixel), a frame rate of 1000 fps can be achieved. As can be seen in Fig. 3b and Supplementary Video 3, the SNR remains sufficient at significantly higher frame rates (SNR ~35 for the action potentials shown in Fig. 3b).

**Figure 4:**
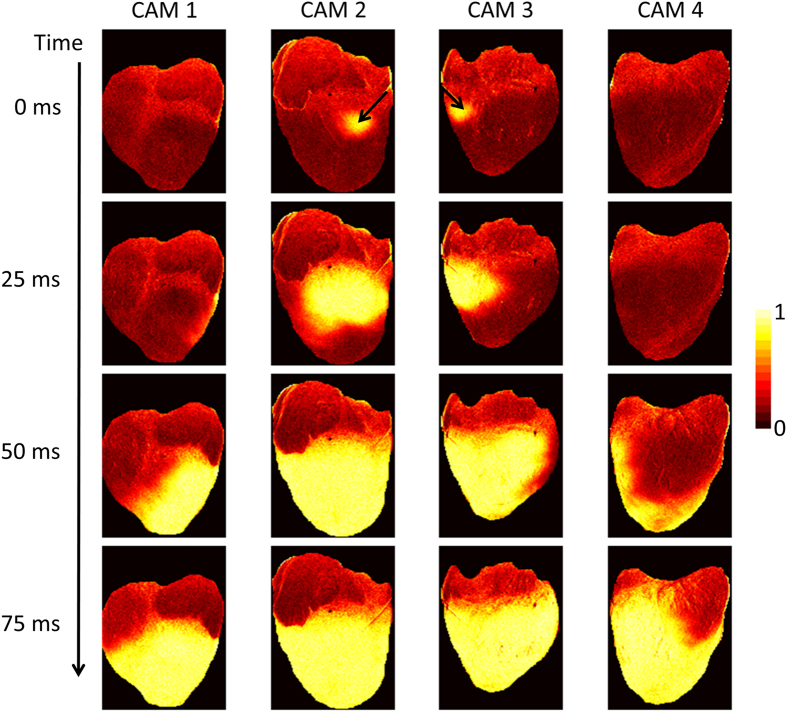
Optical Mapping System 1: Propagation Image Sequences (Langendorff-Perfused Pig Heart). Normalized fluorescence intensity maps (colorbar shown) at progressive time points during electrical pacing (400 ms cycle length). The black arrows indicate the location of electrical point stimulation.

Figure 4 shows a time series of normalized transmembrane voltage fluorescence intensity maps during point electrical pacing (400 ms cycle length). The black arrow shows the stimulation site, which was visible in both CAM 2 and CAM 3 points-of-views. Four frames from a 25 ms interval are shown just after stimulation, illustrating the propagation of the activation front from the stimulation site (Supplementary Video 1). Point electrical stimulation was performed to emphasize the need for panoramic imaging when trying to detect events not visible from only one point-of-view.

### Optical Mapping System 2

**Figure 5:**
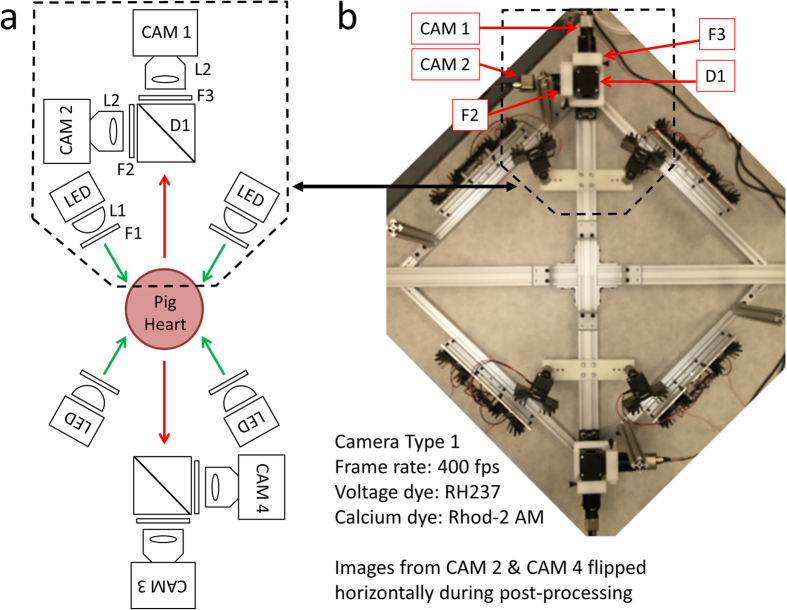
Optical Mapping System 2: Layout (Langendorff-Perfused Pig Heart). (**a**) System schematic showing key components (see text for details). Two cameras and two LED light sources are used to image the heart from each point-of-view (green arrows: excitation light, red arrows: fluorescence emission light). One of the two cameras (CAM 1/CAM 3) is used to image transmembrane voltage while the other (CAM 2/CAM 4) is used to image intracellular free calcium. (**b**) Bird’s-eye view of the imaging and illumination subsystems outlined in (**a**).

In cardiac electrophysiology, transmembrane voltage and intracellular free calcium are two of the most important parameters of interest because of their joint roles in excitation-contraction coupling^17^. As such, the simultaneous measurement of both parameters has become the gold standard in optical mapping. The system shown in Fig. 5 uses the same four high-speed Wide-VGA USB3.0 CMOS cameras to simultaneously image transmembrane voltage and intracellular free calcium from the anterior and posterior surface of the heart. For both points-of-view, one camera images action potentials while another images calcium transients. Each camera (CAM 1 – CAM 4 in Fig. 5a) is configured to record 120 × 160 superpixels (4 × 4 binning mode; 4 × 4 pixels per superpixel) at 400 fps.

We used RH237 and rhod-2AM to measure transmembrane voltage and intracellular free calcium, respectively^18^,^19^,^20^,^21^. These off-the-shelf dyes are widely used in combination by the optical mapping community and have the benefit of being excitable by the same light source. As shown in Fig. 5, two LED light sources are mounted on the left and right side of each dual-camera module. They are identical to the ones used in **Optical Mapping System 1**.

**Figure 6:**
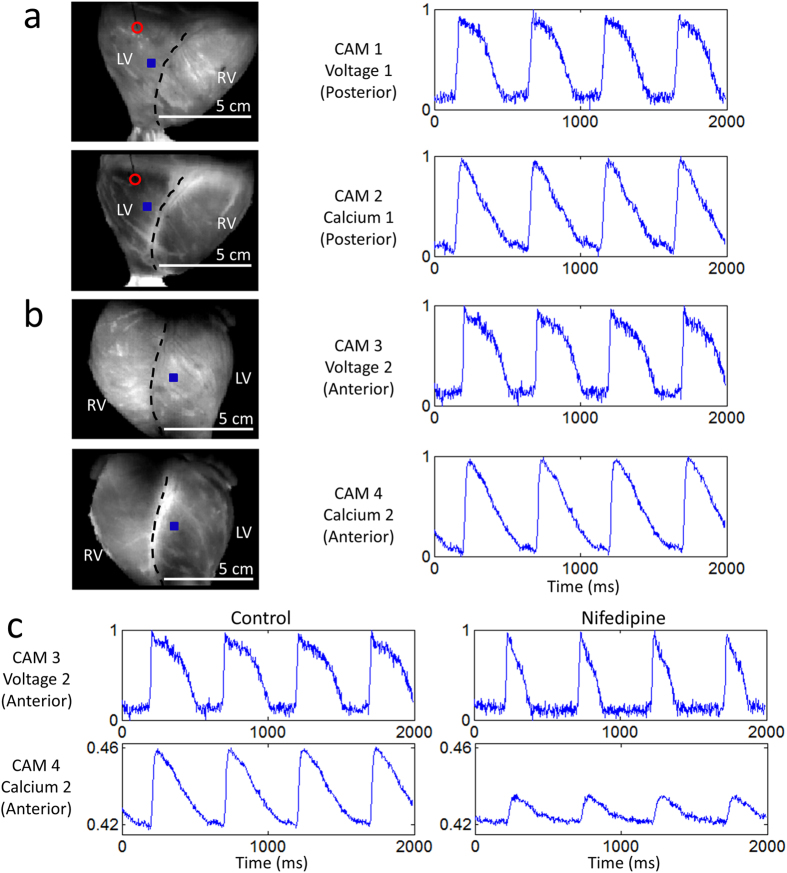
Optical Mapping System 2: Sample Data (Langendorff-Perfused Pig Heart). Normalized fluorescence signals from the (**a**) posterior and (**b**) anterior points-of-view of the heart during electrical pacing, taken from the blue-square regions shown (left panel). In the left panels, the right ventricle and left ventricle are demarcated by black dashed lines and the red circle indicates the location of electrical point stimulation. The four cameras can be independently positioned and rotated to adjust and optimize the position of the heart in their respective fields-of-view. (**c**) Control signals and altered signals (after exposure to 2 μM nifedipine) from a region on the anterior surface of the heart during electrical pacing at 2 Hz (same as in (**b**)). The action potential duration shortened and the calcium transient amplitude decreased. Signals are in arbitrary fluorescence units.

Figure 6 shows sample raw data, again unfiltered in time. The heart was imaged during electrical pacing (Supplementary Video 4) at a cycle length of 500 ms. Compared to di-4-ANEPPS, we found RH237 and rhod-2AM challenging to load in the pig heart, emphasizing the need to optimize dye-loading protocols for each dye and species. The SNRs for CAM 1, CAM 2, CAM 3 and CAM 4 were ~24 (voltage), ~34 (calcium), ~26 (voltage), and ~43 (calcium), respectively, calculated using the signals shown in Fig. 6a,b.

As a proof-of-principle application, we measured the effect of nifedipine on the action potential and calcium transient. Nifedipine is a clinically-relevant 1,4-dihydropyridine calcium-channel blocker that reduces the action potential duration^22^ and calcium transient amplitude^23^. Figure 6c shows sample control signals and signals obtained from the same tissue region ~10 minutes after adding nifedipine to the perfusate (2 μM concentration). The calcium transient signals were not normalized to demonstrate the reduction in signal amplitude.

### Optical Mapping System 3

**Figure 7:**
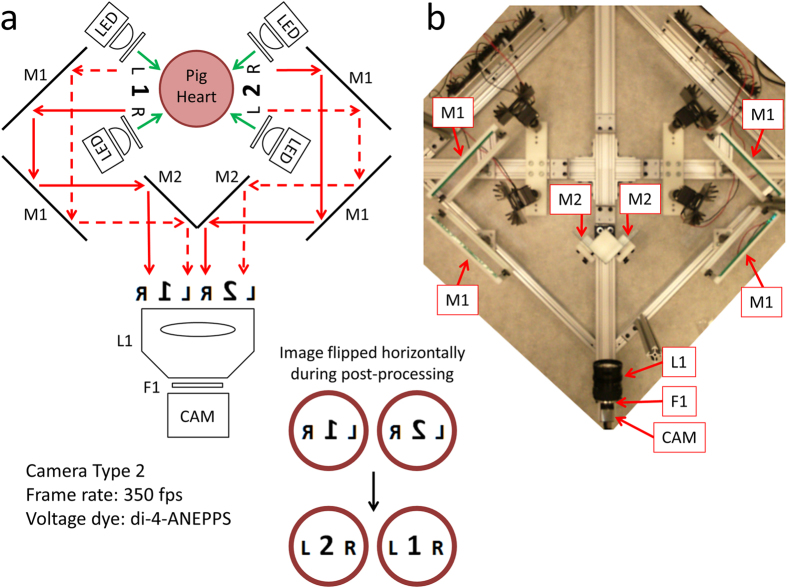
Optical Mapping System 3: Layout (Langendorff-Perfused Pig Heart). (**a**) System schematic showing key components (see text for details). One high resolution camera, six mirrors and four LED light sources are used to image the heart from the anterior and posterior points-of-view (green arrows: excitation light, red arrows: fluorescence emission light). (**b**) Bird’s-eye view of the imaging and illumination subsystems outlined in (**a**).

Recent advances in CMOS camera technologies have made possible high-speed imaging at megapixel spatial resolutions. And with the advent of the high-speed USB3.0 interface, it is possible to continuously record image sequences from cameras directly to the computer’s solid-state drives. The system shown in Fig. 7 takes advantage of the progress in spatial resolution by performing multi-view optical mapping with a single camera. By using six mirrors, the system can image both the anterior and posterior surface of the heart using just one high resolution camera. This system uses a 2.2 Megapixel USB3.0 CMOS camera configured to record 468 × 1024 superpixels (2 × 2 binning mode; 2 × 2 pixels per superpixel) at 350 fps.

Single-camera panoramic optical mapping systems have been constructed and validated before^4^,^24^. These systems used two mirrors placed at the back of the heart to show two reflected images of the posterior surface. This provided three views, spaced ~120° apart, with one direct view and two reflected. Because of the longer light path, reflected images of the heart were smaller. All three views were roughly in focus because of the small size of the hearts used and the large depth-of-focus of the camera lens. The much larger size of the pig hearts used in this study made challenging this implementation, which led us to the presented configuration, which provides two equal views of the anterior and posterior surface. Though less emission light is collected compared to the more direct view approaches of **Optical Mapping Systems 1 and 2**, sufficient signal quality can be achieved with such high-light Langendorff-perfused heart preparations.

**Figure 8:**
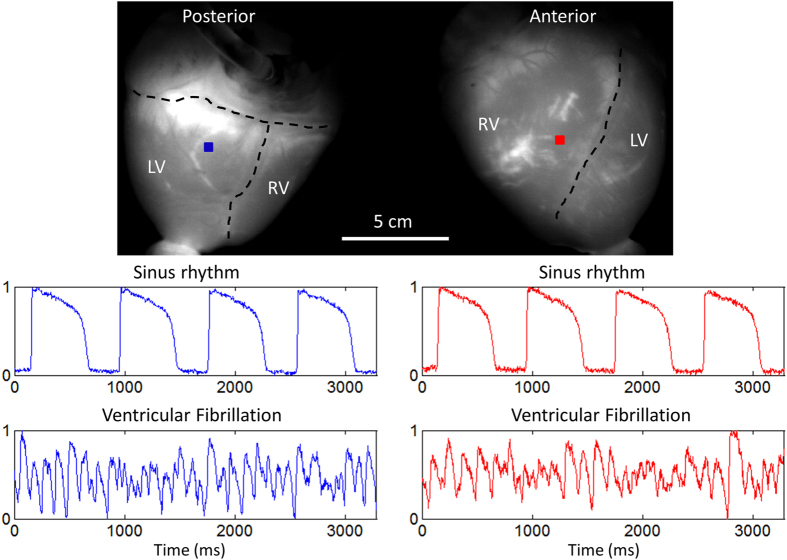
Optical Mapping System 3: Sample Data (Langendorff-Perfused Pig Heart). Normalized fluorescence signals from the posterior and anterior points-of-view of the heart during sinus rhythm and ventricular fibrillation, taken from the blue-square and red-square regions shown, respectively. In the top panel, the right ventricle, left ventricle and atria are demarcated by black dashed lines. Signals are in arbitrary fluorescence units.

Figure 8 shows sample raw data, unfiltered in time. The heart was imaged during sinus rhythm, electrical pacing (Supplementary Video 5) and ventricular fibrillation (Supplementary Video 6). During sinus rhythm, the SNR from points on the posterior (blue) and anterior (red) surface were ~78 and ~55, respectively, calculated using the action potentials shown in Fig. 8.

### A Validation Study in the Langendorff-Perfused Rabbit Heart

An extensive study using a large animal model may be too costly to pursue for many research groups and would certainly be too expensive to use in a biomedical engineering teaching laboratory, for which these systems were also designed for. Therefore, we tested the same CMOS cameras used in **Optical Mapping Systems 1 and 2** in the Langendorff-perfused rabbit heart. Using the uEye Cockpit software, each camera was configured to record 120 × 160 superpixels at 400 fps. For comparison, images were also taken with another camera typically used in optical mapping systems (Evolve 128 EMCCD camera; 128 × 128 pixels; Photometrics, AZ, USA).

**Figure 9:**
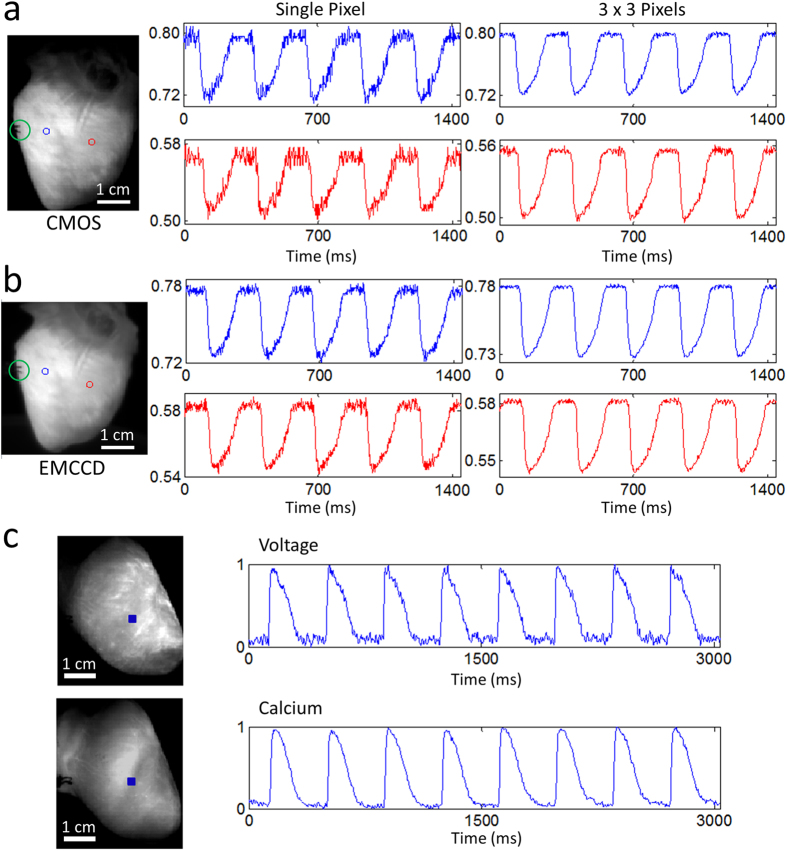
Sample Data from Langendorff-Perfused Rabbit Hearts. Fluorescence signals from two points on the ventricles (left panel; centered on the red and blue circles) during electrical pacing (green circle indicates the location of electrical point stimulation) using the (**a**) CMOS camera and (**b**) EMCCD camera (see text for details). Single pixel and spatially averaged (3 × 3 pixels) traces are shown, unfiltered in time. The SNRs were considerably higher for the EMCCD camera. (**c**) Simultaneous voltage and calcium imaging traces (normalized) taken from the blue-square regions shown (left panel) during sinus rhythm. Signals are in arbitrary fluorescence units.

Figure 9a,b shows sample raw data, unfiltered in time. The traces shown were collected during electrical point stimulation at a 280 ms cycle length. Figure 9a shows data from the CMOS camera while Fig. 9b shows data from the EMCCD camera. The traces were not normalized to illustrate the relative saturation levels. Rabbit hearts were imaged with the CMOS camera while being electrically paced (Supplementary Video 7) and during ventricular fibrillation (Supplementary Video 8). In Fig. 9a, the SNRs for the single-pixel blue and red traces were ~14 and ~9, respectively, while the SNRs for the spatially-averaged (3 × 3 pixels) blue and red traces were ~40 and ~36, respectively. These sample values were calculated using the signals shown in Fig. 9a. For comparison, the same heart was imaged with the EMCCD camera under the same conditions (frame rate = 400 fps and same illumination intensity). In Fig. 9b, the SNRs for the single-pixel blue and red traces were ~23 and ~19, respectively, while the SNRs for the spatially-averaged (3 × 3 pixels) blue and red traces were ~59 and ~47, respectively. These sample values were calculated using the signals shown in Fig. 9b. As expected, the EMCCD camera performed considerably better than the low-cost CMOS camera, and the difference in performance would be magnified in low-light level preparations like cardiac monolayers. However, for high-light level preparations, these modern low-cost CMOS cameras represent inexpensive alternatives capable of yielding data of sufficient quality. Furthermore, Fig. 9c shows sample raw data, again unfiltered in time, demonstrating the suitability of using these CMOS cameras for intracellular calcium imaging in Langendorff-perfused rabbit hearts.

## Discussion

We have described and tested three low-cost optical mapping systems capable of panoramic imaging in a translational heart model. Our most costly implementation has a total system cost less than that of one standard optical mapping camera system, making this method financially viable to more research groups. There are, however, certain limitations with our approach. First, the high-speed CMOS cameras used have lower light sensitivity and more noise compared with the cameras typically used by the optical mapping community (Fig. 9a,b). Thus, using these cameras in low-light level preparations like cardiac monolayers or tissue-engineered constructs may yield unacceptably low SNRs. But for isolated hearts from adult rabbits to adult pigs, we have demonstrated that these low-cost CMOS cameras produce sufficient SNRs to study arrhythmias and drug-action. Second, we used blebbistatin as an excitation-contraction uncoupler to minimize motion ‘artifacts’ in our fluorescence signals. Though this is currently standard practice in the optical mapping field, recent studies have shown that this compound can have deleterious effects on cardiac tissue^25^,^26^. Consequently, it would be desirable to develop a low-cost optical analog to the epicardial electrode sock, designed to record epicardial activity in a beating heart^27^.

Although the presented systems can be used as is, there is still room for improvement. First, dye loading optimization in large animal models, like the pig, is necessary to improve SNRs and to further reduce costs since larger hearts generally require more dye. The use of newly-developed potentiometric dyes will also improve SNRs because they have been shown to produce larger fractional changes in fluorescence during action potentials^24^,^28^,^29^. Second, exploration of other low-cost high-speed cameras is essential as the development of digital industrial cameras has been progressing at a rapid pace. We utilized CMOS cameras made by IDS Imaging Development Systems GmbH because of their cost, performance and programming interface. We are currently exploring newer, and in some cases lower-cost, cameras from the same manufacturer and other manufacturers like Allied Vision Technologies GmbH and Basler AG.

Studying cardiac arrhythmias in animal models has been essential to furthering our understanding of this complex biological phenomenon. However, small animal models like the mouse do not represent a clinically-relevant model for studying either ventricular or atrial fibrillation, especially if the study of arrhythmia dynamics in isolated hearts is complemented with the *in vivo* phenotype. The use of animal models like the goat, sheep or pig permit researchers to obtain *in vivo* phenotypes of arrhythmias similar to those observed in the clinical setting^30^,^31^. Experiments in such models substantially increase the cost of both *in vivo* and *ex vivo* studies^32^. As such, low-cost implementations of powerful experimental methods like panoramic optical mapping will help enable more extensive *in vivo* and *ex vivo* studies in large mammals with complex arrhythmias, a capacity limited to few laboratories. Importantly, the panoramic mapping approach enables one to track rotors on the myocardial surface at a resolution not achievable in current clinical electrophysiology procedures, which are limited to simultaneous multipolar recordings^33^,^34^.

By lowering the financial threshold for implementing panoramic optical mapping, building a system that combines panoramic optical mapping with electrical mapping may be feasible while maintaining cost containment. For instance, simultaneous panoramic imaging and endocardial mapping using a balloon electrode array would provide both epicardial and endocardial information, respectively, which are both relevant in the study of ventricular arrhythmias^35^,^36^,^37^. Furthermore, doing both of these measurements in a torso-tank fitted with hundreds of body-surface electrodes would simultaneously provide a complete set of electrocardiogram information on the pseudo-torso surface^38^,^39^. ECG imaging based on body surface potentials allows clinicians to non-invasively infer the electrical activity in the heart by solving an “inverse problem.” Though much progress has been made in reconstructing cardiac electrical activity from these remote electrode measurements, validating these inferences with combined panoramic optical mapping and endocardial mapping should prove useful. Finally, parallel work in the field of whole-heart computational modeling will undoubtedly benefit from such multi-modal validation measurements, leading to a more coherent picture of complex arrhythmias and therapeutic interventions to restore sinus rhythm^3^,^40^,^41^.

## Materials and Methods

### Computer System

We built a custom computer to support all three optical mapping systems (Figure S1 in Supplementary Information). The computer is composed of the following key components: 1) operating system: Microsoft Windows 7 Professional 64-bit, 2) processor: Intel Core i7-3770 Ivy Bridge Quad-Core 3.4 GHz, 3) motherboard: ASUS P8Z77-V LK, 4) memory: G.SKILL 16 GB (2 × 8 GB) 240-Pin DDR3 SDRAM and 5) three solid state drives (SSDs): SAMSUNG 850 PRO 2.5″ 128 GB SSD. One SSD is used for the operating system and the other two are used for saving image sequences from the cameras in real time. All computer components were purchased from Newegg (Newegg Canada Inc., Richmond Hill, Canada). In addition, the computer uses a 4-port USB3.0 PCI express card (part #: AL00014; IDS Imaging Development Systems GmbH, Obersulm, Germany) for camera communication and data acquisition. To record the frame exposure start signals from each camera, we use a PC oscilloscope (PicoScope 2205 MSO; Pico Technology Limited, Cambridgeshire, United Kingdom). Time stamps for image frames received by the computer are also recorded by the acquisition software.

### Software

Camera configuration, image alignment and focusing are performed using uEye Cockpit, a component of the downloadable IDS Software Suite (IDS Imaging Development Systems GmbH). Custom acquisition software for the cameras was written in the C# programming language using the IDS Software Suite. The light sources are controlled by a microcontroller-based circuit, which can communicate with the computer via the USB interface (Figure S1 in Supplementary Information). Custom control and communication software for the microcontroller and computer were written in Spin (Parallax Inc., Rocklin, CA) and MATLAB (The MathWorks Inc., Natick, MA), respectively, and are described elsewhere^42^. Custom software written in MATLAB was used for image processing.

Authors P.L. or D.F.R. can be contacted for a copy of the software.

### Mechanical Framework

The design and machining of the mechanical framework for the optical mapping systems were done in-house. Mechanical and optomechanical components were purchased from McMaster-Carr (Elmhurst, IL) and Thorlabs Inc. (Newton, NJ). Raw metal, such as aluminum bars and rods, were acquired from a local metal distributor (Mr. Metal, Etobicoke, Canada).

### Electronics

The major electronic components of the system are outlined in Figure S1 (Supplementary Information). The custom-built illumination controller, which drives all the LED light sources, is a modification of a system described before^42^, where detailed circuit diagrams and software code are provided. The primary difference is in the total light output power supported by the controller. Because of the large surface area of the heart and the need to drive up to eight high-power LEDs simultaneously, a significantly more powerful controller was designed and built. All electronic components, including the LEDs, can be acquired from major electronic components distributors (Digi-Key Electronics, Thief River Falls, MN; Mouser Electronics Inc., Mansfield, TX).

Authors P.L. or D.F.R. can be contacted for details on the electronics.

### Optical Mapping System 1

As shown in Fig. 1, this system uses four identical CMOS cameras CAM 1 – CAM 4 (part #: UI-3220CP-M-GL; IDS Imaging Development Systems GmbH) to acquire images from four points-of-view spaced 90° apart. Between 500 nm – 800 nm, the cameras have a quantum efficiency of 40–50% (datasheets available on the company website). Using the uEye Cockpit software, each camera is configured to record 120 × 160 superpixels (4 × 4 binning mode; 4 × 4 pixels per superpixel) at 400 fps. All four cameras are triggered to start image acquisition simultaneously via software, after which they run in freerun mode to achieve maximum frame rates (i.e. exposure and readout-transfer of the image data are performed in parallel).

Eight green LEDs (part #: CBT-90 Green; Luminus Devices Inc., Woburn, MA) are used to excite di-4-ANEPPS dye-loaded tissue (Fig. 1a). The excitation light from each LED passes through a plano-convex lens L1 (part #: LA1951; Thorlabs Inc.) and excitation filter F1 (part #: FF01-534/20-25; Semrock Inc., Rochester, NY). Fluorescence emission light from the tissue is passed through a custom-made emission filter F2 (part #: ET577.5LP; Chroma Technology Corp, Bellows Falls, VT) and then collected with a camera lens L2 (part #: DO-1795; Navitar Inc., Rochester, NY).

For the 1 kHz recordings shown in Fig. 3b, another CMOS camera was used (part #: UI-3360CP-NIR-GL; IDS Imaging Development Systems GmbH). Between 500 nm – 800 nm, the camera has a quantum efficiency of 40–70% (datasheets available on the company website).This higher resolution camera was configured to record 160 × 220 superpixels (4 × 4 binning mode; 4 × 4 pixels per superpixel) at 1000 fps. The same emission filter and camera lens were used in this proof-of-principle recording.

### Optical Mapping System 2

As shown in Fig. 5, this system uses four identical CMOS cameras CAM 1 – CAM 4 (part #: UI-3220CP-M-GL; IDS Imaging Development Systems GmbH) to acquire images from the anterior and posterior surface of the heart. Each camera is configured the same way as in **Optical Mapping System 1**.

Four green LEDs (part #: CBT-90 Green; Luminus Devices Inc., Woburn, MA) are used to excite RH237 and rhod-2AM dye-loaded tissue (Fig. 5a). The excitation light from each LED passes through a plano-convex lens L1 (part #: LA1951; Thorlabs Inc.) and excitation filter F1 (part #: FF01-534/20-25; Semrock Inc., Rochester, NY). Fluorescence emission from both dyes are first separated by a dichroic mirror D1 (part #: T685LPXR; Chroma Technology Corp). Separated emission light from rhod-2AM and RH237 then pass through emission filter F2 (part #: ET590/50 M; Chroma Technology Corp) and custom emission filter F3 (part #: ET700LP; Chroma Technology Corp), respectively. Both filtered lights are then collected by camera lenses, both labeled L2 (part #: DO-1795; Navitar Inc.). And because of the reflections at the dichroic mirrors, images from CAM 2 and CAM 4 are flipped horizontally during image processing.

### Optical Mapping System 3

As shown in Fig. 7, this system uses one CMOS camera CAM (part #: UI-3360CP-NIR-GL; IDS Imaging Development Systems GmbH) and six mirrors to acquire images from the anterior and posterior surface of the heart. Using the uEye Cockpit software, the camera is configured to record 468 × 1024 superpixels (2 × 2 binning mode; 2 × 2 pixels per superpixel) at 350 fps. For both sides of the heart, fluorescence emission is reflected by two 127 × 178 mm flat mirrors, both labeled M1 (part #: 40-043; Edmund Optics Inc., Barrington, NJ, USA), and one 81 × 100 mm flat mirror M2 (part #: 41-621; Edmund Optics Inc.). Reflected emission light from both sides are then collected by a camera lens L1 (part #: DO-5095; Navitar Inc.) and filtered with custom emission filter F1 (part #: ET577.5LP; Chroma Technology Corp). Images are flipped horizontally during image processing because of the mirror reflections.

### Langendorff-Perfused Pig Heart

Six Large White pigs weighing 17–20 kg were used in this proof-of-principle study (n = 2 for **Optical Mapping System 1**; n = 2 for **Optical Mapping System 2**; n = 2 for **Optical Mapping System 3**). The experimental studies were conducted in accordance with institutional guidelines and regulations [National (ECC/566/2015, RD53/2013) and European (2010/63/EU) guidelines for the care and use of laboratory animals]. All *in vivo* experimental procedures were evaluated and granted by the Institutional Animal Care and Use Committee (IACUC) of CNIC and the Local Competent Authority. Surgical procedures were carried under general anesthesia following premedication with a combination of intramuscular Xylazine (2 mg/kg i.m.) and Midazolam (0.5 mg/kg i.m.) and induced with intravenous Ketamine (20 mg/kg i.v.). After intubation and venous catheterization through the marginal vein, animals were mechanically ventilated with intermittent positive pressure and anesthesia was maintained by a combination of Fentanyl (0.010 mg/kg/h i.v.) and Sevoflurane (2%). Heparin (300 UI/kg) was administered to avoid coronary blood coagulation and vital signs were monitored during the procedure to control anesthesia status. Hearts were exposed via median sternotomy and a 9 VDC battery was used to induce ventricular fibrillation during the extraction. Excised hearts were then submerged in cold (4 °C) Tyrode’s solution, cleaned and then cannulised through the aorta and connected to a constant-flow Langendorff-perfusion apparatus. Hearts were perfused with oxygenated (95% O_2_, 5% CO_2_) Tyrode’s solution (composition in mM: NaCl 130, NaHCO_3_ 24, NaH_2_PO_4_ 1.2, MgCl_2_ 1, Glucose 5.6, KCl 4, CaCl_2_ 1.8 and albumin 0.04 g/L) at a circulating flow rate of 200–240 mL/min^43^. Ionic pH of the perfusate (7.4), oxygenator status and temperature (36.5–37.5 °C) were monitored during the experiment. Intracavitary volumes in atrial and ventricular chambers were adjusted to resemble physiological stretch in diastole as described elsewhere^44^.

Fluorescent dyes were injected at a short distance upstream from the aortic cannula for coronary perfusion. For transmembrane voltage imaging, hearts were loaded with dye by slowly delivering, without recirculation, 0.250 mL of 2.5 mg/mL (in DMSO) of either di-4-ANNEPS (part #: 61010; Biotium Inc., Hayward, CA) or RH237 (part #: 61018; Biotium Inc.). For intracellular free calcium imaging, hearts were loaded with dye by slowly delivering 2 mL of 1 mg/mL (in DMSO) rhod-2AM (part #: 50024; Biotium Inc.). During calcium dye loading, the heart underwent a recirculating perfusion of 1.5 L of perfusate containing 0.5 mM probenecid (part #: 2522; TEFLabs Inc., Austin, TX) for 30 minutes. After dye loading, the perfusate was replaced with fresh perfusate containing 10 μM blebbistatin (part #: 674289–55–5; Cayman Chemical Company, Ann Arbor, MI) to reduce contraction during imaging. For drug testing, Nifedipine (N7634; Sigma-Aldrich Dorset, UK) from a 20 mM stock solution (dissolved in DMSO) was diluted in the perfusate to 2 μM.

### Langendorff-Perfused Rabbit Heart

Female New Zealand White rabbits (body weight ~3 kg, n = 4) were used in this supplementary study. The experimental studies were conducted in accordance with institutional guidelines and regulations [National (ECC/566/2015, RD53/2013) and European (2010/63/EU) guidelines for the care and use of laboratory animals]. All *in vivo* experimental procedures were evaluated and granted by the Institutional Animal Care and Use Committee (IACUC) of CNIC and the Local Competent Authority. Surgical procedures were carried under general anesthesia following premedication with a combination of intramuscular Xylazine (10 mg/kg i.m.) and Midazolam (2 mg/kg i.m.) and induced with a slow intravenous administration of Ketamine (20 mg/kg i.v.). After intubation and venous catheterization through the marginal vein, animals were mechanically ventilated with intermittent positive pressure and anesthesia was maintained by a combination of Fentanyl (0.010 mg/kg/h i.v.) and Sevoflurane (2%). Heparin (300 UI/kg) was administered to avoid coronary blood coagulation and vital signs were monitored during the procedure to control anesthesia status. Excised hearts were then submerged in cold (4 °C) Tyrode’s solution, cleaned and then cannulised through the aorta and connected to a constant-flow Langendorff-perfusion apparatus. Hearts were perfused with oxygenated (95% O_2_, 5% CO_2_) bicarbonate-buffered solution (composition in mM: NaCl 123, CaCl_2_ 1.8, KCl 5.4, MgCl_2_ 1.2, NaH_2_PO_4_ 1.4, NaHCO_3_ 24, Glucose 10) at a circulating flow rate of 30 mL/min. Ionic pH of the perfusate (7.4) and temperature (36–37 °C) were monitored during the experiment.

Fluorescent dyes were injected at a short distance upstream from the aortic cannula for coronary perfusion. For transmembrane voltage imaging, hearts were loaded with dye by slowly delivering, without recirculation, either 1) a solution containing 20 μL of di-4-ANBDQPQ (27 mmol/L in ethanol, University of Connecticut Health Center, USA) and 2 μL of Pluronic F-127 (20% stock solution in DMSO; part #: 2510; TEFLabs Inc., Austin, TX) or 2) 20 μL of 2.5 mg/mL (in DMSO) RH237 (part #: 61018; Biotium Inc., Hayward, CA). For intracellular free calcium imaging, hearts were loaded with dye by slowly delivering 250 μL of 1 mg/mL (in DMSO) rhod-2AM (part #: 50024; Biotium Inc.). During calcium dye loading, the heart underwent a recirculating perfusion of 300 mL of perfusate containing 0.5 mM probenecid (part #: 2522; TEFLabs Inc.) for 30 minutes. After dye loading, the perfusate was replaced with fresh perfusate containing 10 μM blebbistatin (part #: 674289-55-5; Cayman Chemical Company, Ann Arbor, MI) to reduce contraction during imaging.

### Optical Mapping in the Rabbit Heart

For imaging the voltage dye di-4-ANBDQPQ, two red LEDs (part #: CBT-90 Red; Luminus Devices Inc., Woburn, MA) were used to excite dye-loaded tissue (Fig. 9a,b). The excitation light from each LED was passed through a plano-convex lens (part #: LA1951; Thorlabs Inc., Newton, NJ) and excitation filter (part #: ZET642/20X; Chroma Technology Corp, Bellows Falls, VT). Fluorescence emission light from the tissue was passed through a custom-made emission filter (part #: ET700LP; Chroma Technology Corp) and then collected with a camera lens (part #: DO-1795; Navitar Inc., Rochester, NY). The same emission filter and camera lens were used for both the CMOS and EMCCD camera. For imaging voltage dye RH237 and calcium dye rhod-2AM, the same configuration as **Optical Mapping System 2** was used.

## Additional Information

**How to cite this article**: Lee, P. *et al*. Low-Cost Optical Mapping Systems for Panoramic Imaging of Complex Arrhythmias and Drug-Action in Translational Heart Models. *Sci. Rep.* **7**, 43217; doi: 10.1038/srep43217 (2017).

**Publisher's note:** Springer Nature remains neutral with regard to jurisdictional claims in published maps and institutional affiliations.

## Supplementary Material

Supplementary Information

Supplementary Video 1

Supplementary Video 2

Supplementary Video 3

Supplementary Video 4

Supplementary Video 5

Supplementary Video 6

Supplementary Video 7

Supplementary Video 8
