# Supplementary material for: Low-Cost Optical Mapping Systems for Panoramic Imaging of Complex Arrhythmias and Drug-Action in Translational Heart Models
Source: Sci Rep. 2017 Feb 27;7:43217. doi: 10.1038/srep43217 (PMC5327492; doi:10.1038/srep43217)
Supplement: Supplementary Information [file srep43217-s1.pdf]

Low-Cost Optical Mapping Systems for Panoramic Imaging of Complex Arrhythmias and Drug-Action in Translational Heart Models

Peter Lee[1]
Conrado J. Calvo[2, 3, 4, 5]
José M. Alfonso-Almazán[3]
Jorge G. Quintanilla[3, 5, 6]
Francisco J. Chorro[4, 5]
Ping Yan[7]
Leslie M. Loew[7]
David Filgueiras-Rama[3, 5, 6]
José Millet[2, 5]

[1] Essel Research and Development Inc., Toronto, Canada
[2] ITACA, Universitat Politècnica de València, Valencia, Spain
[3] Spanish National Cardiovascular Research Center (CNIC), Madrid, Spain
[4] Departments of Physiology and Cardiology, Universitat de València, Clinic Hospital - INCLIVA, Valencia, Spain
[5] Centro de Investigación Biomédica en Red de Enfermedades Cardiovasculares (CIBERCV), Spain
[6] Instituto de Investigación Sanitaria del Hospital Clínico San Carlos (IdISSC), Madrid, Spain
[7] Richard D. Berlin Center for Cell Analysis and Modeling, University of Connecticut Health Center, Connecticut, USA

Supplementary Figures

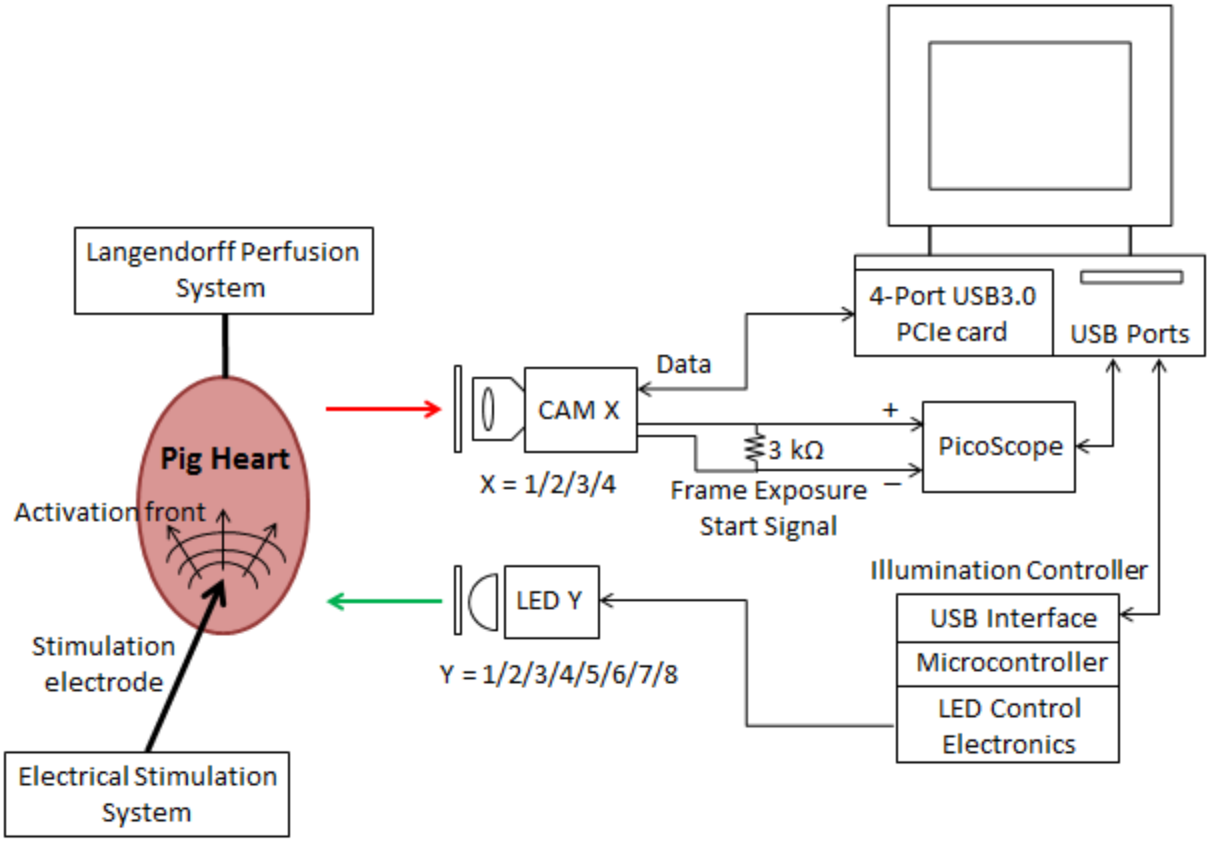

Figure S1: Optical Mapping System Layout
System schematic showing the major components of the optical mapping systems (see text for details). The system can support up to four cameras and eight high-power LED light sources.

Supplementary Videos

Supplementary Video 1.mp4
Optical Mapping System 1 (Langendorff-Perfused Pig Heart): Normalized transmembrane voltage fluorescence intensity movies from all four cameras during electrical pacing (400 ms cycle length), illustrating the propagation of the activation front from the stimulation site.

Supplementary Video 2.mp4
Optical Mapping System 1 (Langendorff-Perfused Pig Heart): Normalized transmembrane voltage fluorescence intensity movies from all four cameras during ventricular fibrillation.

Supplementary Video 3.mp4
Langendorff-Perfused Pig Heart: Normalized transmembrane voltage fluorescence intensity movie from one camera at 1000 fps, showing an electrically paced activation followed by activation under sinus rhythm. Epicardial breakthrough sites, one on the right and one on the left ventricle, are clearly visible during sinus rhythm.

Supplementary Video 4.mp4
Optical Mapping System 2 (Langendorff-Perfused Pig Heart): Normalized transmembrane voltage and intracellular free calcium fluorescence intensity movies during electrical pacing (500 ms cycle length), illustrating the propagation of the activation front from the stimulation site. The delay of the calcium transient to action potential peak can be seen.

Supplementary Video 5.mp4
Optical Mapping System 3 (Langendorff-Perfused Pig Heart): Normalized transmembrane voltage fluorescence intensity movies during rapid electrical pacing (250 ms cycle length), illustrating the propagation of the activation front from the stimulation site.

Supplementary Video 6.mp4
Optical Mapping System 3 (Langendorff-Perfused Pig Heart): Normalized transmembrane voltage fluorescence intensity movies during ventricular fibrillation.

Supplementary Video 7.mp4
Langendorff-Perfused Rabbit Heart Optical Mapping: Normalized transmembrane voltage fluorescence intensity movie from the epicardium of an isolated rabbit whole-heart during electrical pacing (280 ms cycle length), illustrating the propagation of the activation front from the stimulation site.

Supplementary Video 8.mp4
Langendorff-Perfused Rabbit Heart Optical Mapping: Normalized transmembrane voltage fluorescence intensity movie from the epicardium of an isolated rabbit whole-heart during ventricular fibrillation, induced by the application of a 9-V battery to the epicardial surface.
